# The temporal-spatial encoding of acupuncture effects in the brain

**DOI:** 10.1186/1744-8069-7-19

**Published:** 2011-03-23

**Authors:** Wei Qin, Lijun Bai, Jianping Dai, Peng Liu, Minghao Dong, Jixin Liu, Jinbo Sun, Kai Yuan, Peng Chen, Baixiao Zhao, Qiyong Gong, Jie Tian, Yijun Liu

**Affiliations:** 1 Medical Image Processing Group, Institute of Automation, Chinese Academy of Sciences, Beijing, 100190, China; 2 Life Sciences Research Center, School of Life Sciences and Technology, Xidian University, Xi'an, Shaanxi 710071, China; 3 Department of Radiology, Beijing Tiantan Hospital, Capital University of Medical Sciences, PR China; 4 Beijing TCM Hospital affiliated to Capital University of Medical Sciences, China; 5 Beijing University of Chinese Medicine, China; 6 Huaxi MR Research Center (HMRRC), Department of Radiology, the Center for Medical Imaging, West China Hospital of Sichuan University, China; 7 Department of Psychiatry and McKnight Brain Institute, University of Florida, Gainesville, FL 32610, USA

## Abstract

**Background:**

Functional acupoint specificity is crucial to the clinical efficacy of acupuncture treatment, such as pain relief. Whether acupuncture needling at a peripheral acupoint produces distinct patterns of brain responses remains controversial.

**Results:**

This fMRI study employed the complex network analysis (CNA) to test the hypothesis that acupuncture stimulation at an acupoint correspondingly induced activity changes in one or more intrinsic or resting-state brain networks. Built upon the sustained effect of acupuncture and its time-varying characteristics, we constructed a dynamic encoding system with the hub anchored at the posterior cingulate cortex and precuneus (PCC/pC). We found that needling at two visual acupoints (GB37 and BL60) and a non-visual acupoint (KI8) induced a spatially converging brain response, which overlapped at the PCC/pC. We also found distinct neural modulations during and after acupoint stimulation. During this period, the PCC/pC interacted with a visual resting-state network in different patterns. Furthermore, there was a delayed functional correspondence between the intrinsic visual network and manipulation over the visual acupoints (i.e., GB37 or BL60), but not the non-visual acupoint (KI8) via the PCC/pC, implicating a specific temporal-spatial encoding/decoding mechanism underlying the post-effect of acupuncture.

**Conclusions:**

This study provided an integrated view exploring the functional specificity of acupuncture in which both the needling sensation and the following neural cascades may contribute to the overall effect of acupuncture through dynamic reconfiguration of complex neural networks.

fMRI, acupoints, posterior cingulate cortex, precuneus, temporal-spatial encoding, resting-state networks

## Background

Acupuncture is practiced worldwide in clinical use to treat a wide range of diseases, particularly in pain management [1-5]. However, acupuncture has not gained a proper niche in modern biomedical disciplines [1,6,7]; this is partially due to the fact that physiological mechanisms underlying acupuncture effects still remain unidentified [8]. Among all research interests, the central representations of peripheral acupuncture stimulation with regard to its functional specificity are most appealing [9-11]. Although supporting evidence from a number of neuroimaging studies exists [8,11-18], the debate over acupoint-brain specificity continues [19-21].

Functional magnetic resonance imaging (fMRI) studies consistently demonstrated that brain activations were evident during the stimulation of visual function-associated acupoints regardless of acupuncture modalities and stimulating intensities [22,23]. However, most previous findings disputing acupoint specificity were attributed to its spatial distribution [10,24]. Most notably, Cho et al [25] withdrew their earlier agreement with acupoint specificities, in which a correspondence between the manipulation over visual acupoints and distinct activation patterns in the occipital lobe was reported [13]. The controversial results, however, could have been due to incorrect conceptualization about the acupuncture effects and inadequate methods used for studying neural substrates underlying acupoint specificity [6,11,26].

A large proportion of previous fMRI studies employed a "BLOCK" experimental design, which is built on a stimulation-response model (called the "On-Off" model) with the assumption that the BOLD signal should instantaneously fall back to its initial (pre-stimuli) status after stimulation. Our studies indicated that the BLOCK design and related analysis methods bias the experimental results [8,11,18]. The instantaneous cerebral responses during acupuncture needling using a blocked paradigm may have been derived mainly from sensation activities but not their functional effects specific to healing. Such functional imaging results based upon subjective constraints may not be able to characterize acupuncture specificities. Acupuncture has been shown to manipulate sustained influence over the brain even after cessation of the needling stimulation [11,27,28], suggesting that acupuncture effects have a time-variant feature at each functional brain region mapped after the needling manipulation and may be acupoint-specific [8,14].

On the other hand, stimulating different acupoints for treating various clinical conditions is usually accompanied with multi-dimensional physiological as well as psychological responses, indicating that the peripheral acupoint-brain interaction may engage complex neural substrates [29,30]. The brain regions are organized into interleaved networks to accomplish various functions or healing effects when a neural intervention is triggered. This suggests that specific brain networks may facilitate a correspondence between acupuncture stimulation and the central nervous system (CNS). The encoding by the CNS responses to peripheral stimulation at different acupoints is then deciphered in a functional specific network. Furthermore, the late sustained response mobilizes the brain networks to implement certain functions. However, former research overly focuses on the role of discrete brain regions and has ignored the underlying integration of these dynamic neural processes. Therefore, previous studies focusing on *Deqi *failed to reveal acupuncture specificities [31]; the link between peripheral acupoints and the clinical effects of acupuncture is still missing.

**Figure 1:**
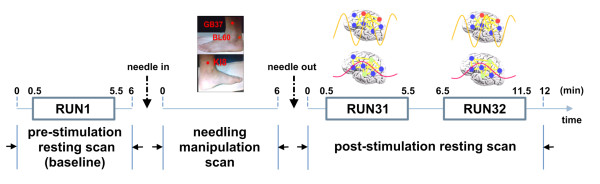
**Experimental paradigm and the schematic of the current research frame**. There are three scan sessions: a pre-acupuncture resting state (baseline) scan lasting 6 minutes; a needling stimulation scan lasting 6 minutes with a single trail electroacupuncture (EA) stimulation; and a post-needling scan lasting 12 minutes. Datasets RUN1, RUN31 and RUN32 are truncated from the pre-acupuncture resting state and the post-needling scan (see the **Methods **for details). This paper focuses on the the delayed post effects of acupuncture, which refers to the post-needling scan. Therefore, datasets RUN1, RUN31 and RUN32 are used for further analysis.

The research on acupuncture specificities needs to be based on a new framework by considering acupuncture's long lasting effects [28]. We propose that acupuncture effects have two phases, i.e. the early acute effects, which involve the instant cerebral response to needling manipulation, and the delayed long-last effects (Figure 1). With particular interest in the post-effects after stimulating different acupoints, we took a dynamic approach by taking into account the temporal processes (before, during, and post-needling) and the contrast between these phases (Figure 1). To verify the aforementioned assumptions and identify acupoint specificities which may correspond to acupuncture's therapeutic specificities, our research group used a non-repeated event-related (NRER) paradigm [17] and employed two visual acupoints (GB37 and BL60) and a non-visual acupoint (KI8) in the current fMRI experiment. We hypothesized that acupuncture stimulation at a specified acupoint would induce corresponding brain activity changes in one or more specified intrinsic or resting-state networks (RSNs) [32,33]. To test our hypothesis, the discrete cosine transform (DCT) [34] and conjunction analysis [15] methods were utilized to determine a collective functional modulation pattern of these networks after needling. Furthermore, a graphic theory-based complex network analysis (CNA) approach [35-39] was introduced to reveal how specific neural responses interacted with stimulation at different acupoints.

## Results

### Psychological results

**Figure 2:**
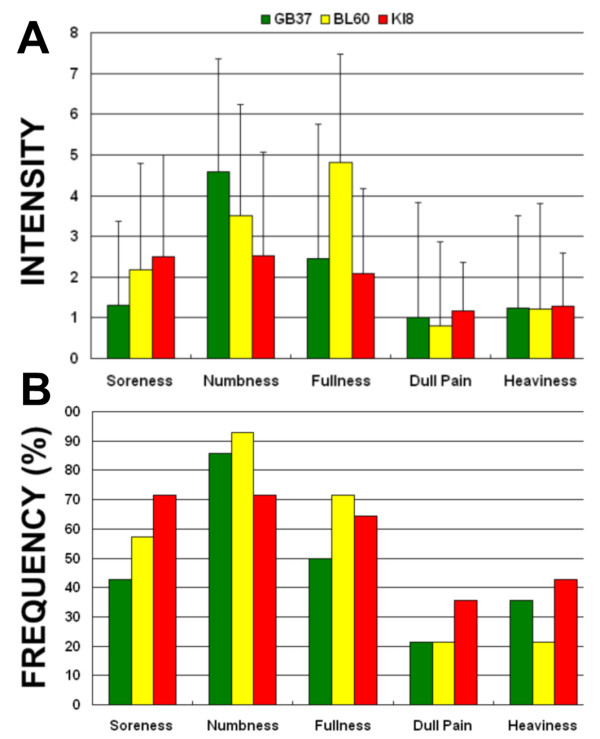
**Results of psychophysical *Deqi *sensations**. A. The intensity of the reported sensations measured by an average score (with standard error bars) on a scale from 0 denoting no sensation to 10 denoting an unbearable sensation. The average stimulus intensities were approximately similar during acupuncture at GB37, BL60 and KI8. No statistical differences were found among groups with regards to each sensation (p < 0.05). B. The percentage of subjects who reported having experienced the given sensation (at least one subject experienced the seven sensations listed). Numbness, fullness, dull pain, heaviness and soreness were found no statistical differences for all three groups (p < 0.05).

All volunteers kept awake according to their reports after scanning. Consistent with the previous findings[9,40], all our subjects experienced various degrees of the needling-associated sensations when the peripheral acupoints were stimulated. The prevalence of Deqi sensations was expressed as the percentage of individuals in the group that reported the given sensations. The average stimulus intensities were approximately similar during acupuncture at GB37, BL60 and KI8. No statistical differences were found among groups with regards to each sensation (p > 0.05) (Figure 2). Numbness, fullness, dull pain, heaviness and soreness were found no statistical differences for all three groups (p > 0.05). These results indicated that acupuncture at non-acupoint with same needling manipulation could effectively reduce the subjects' bias toward the stimulation. Additionally, no subjects reported the experience of sharp pain during the experiment.

### DCT and conjunction analysis results

**Figure 3:**
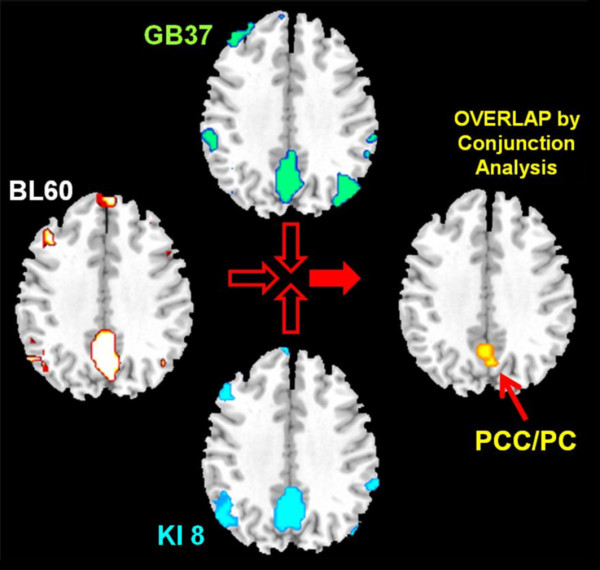
**Results of conjunction analysis based on the DCT results**. The masks of group-level DCT results of all three groups, i.e. GB37, BL60 and KI 8, were used to generate this map. The only overlapped region located in the posterior cingulate cortex and precuneus (PCC/pC) (*p *< 0.001, corrected, cluster size > 20 voxels, see the **Methods **for details).

The results revealed that the overlapping brain regions were predominantly located in the PCC/pC (Figure 3). This finding is not coincidental. Previous acupuncture studies indicated that the PCC/pC is frequently reported during acupuncture stimulation (18, 34, 35). Our results also depicted this region as a general area of non-specific effects of acupoint needling. Previous studies with regards to the resting state detected that the PCC/pC played a pivotal role in the default mode network (33) with higher metabolism consumption and stronger spontaneous signal changes in this region (25, 30). This finding further implicated that the PCC/pC might be an important brain region involved in sustained modulations of acupuncture effects.

### Functional connectivity analysis results

**Figure 4:**
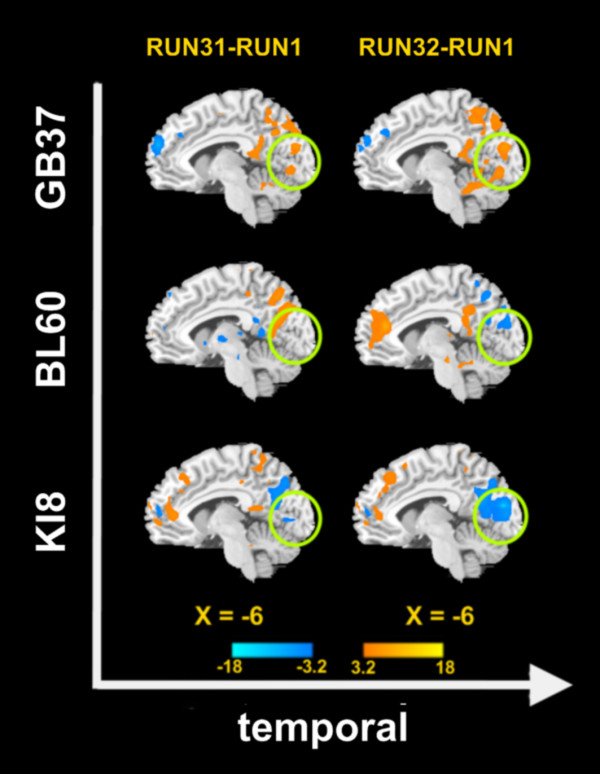
**Contrasting results between post-acupuncture functional connectivity and pre-acupuncture functional connectivity for three groups**. The voxel-wised functional connectivity analysis was conducted using the PCC/pC as a seed point. The paired *t*-test compared the sustained effects with the baseline, i.e, RUN 31 v.s. RUN1 and RUN 32 v.s. RUN1) and the results were thresholded at *P *< 0.005 (uncorrected) and the cluster size > 5 voxels. Warm (cold) colors indicated the increase (decrease) in functional connectivity.

Using the PCC/pC as the ROI, we conducted a voxel-wise functional connectivity analysis [33,41,42] and used group paired *t*-tests to compare the results between the baseline state and sustained phases of the acupuncture effects (Figure 4). Since our main focus was on the visual areas, only changes in the occipital lobe were detected. Three distinct patterns of functional connectivity were observed in the visual cortex (circles) with respect to the three acupoints. There were significant increases in the functional connectivity following stimulation at the two visual acupoints (GB37 and BL60). However, functional connectivity in the occipital cortex was decreased after stimulating the non-visual acupoint (KI8). Moreover, such differences in the visual connectivity remained over the entire late phase (i.e., across Run 31 and Run 32) (Figure 4).

**Figure 5:**
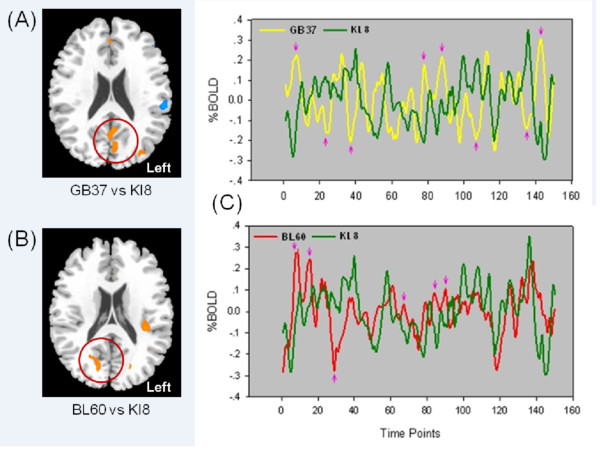
**Illustration of variations of modulating effect of acupuncture over different acupoints**. Comparisons of the functional connectivity were conducted between the visual acupoint group (GB37 or BL60) and the non-visual acupoint group (KI 8) during the late phase of acupuncture (Run 3): (**A**) GB37 vs. KI 8; (**B**) BL60 vs. KI8. The two sample *t*-test were used to determine significant between-group differences (*P *< 0.005 with a cluster size of 5 voxels). (**C**) The averaged time courses of fMRI BOLD signals in the occipital lobe, where showed significant between-group differences (circles on **A **and **B**). The temporal responses revealed almost opposite response patterns after stimulations over visual acupoint and the non-visual acupoint, i.e. GB37 vs. KI 8 and BL60 vs. KI8. The correlation coefficient between GB37 and KI 8 was -0.35 (P < 0.05, Bonferroni correction) and that of BL60 vs. KI8 was -0.18 (P < 0.05, Bonferroni correction).

A direct comparison between the functional connectivity of the GB37 group and that of the KI8 group yielded significantly positive correlations with the occipital lobe during the late phase of acupuncture (Figure 5a), indicating a functional distinction between these two acupoints (GB37 vs. KI8). A similar contrast (BL60 vs. KI8) was also found (Figure 5b). Interestingly, the temporal responses of the same occipital area exhibited almost opposite changes in the BOLD signal for the visual acupoints (GB37 or BL60) and the non-visual acupoint (KI8) (Figure 5c). More importantly, even for GB37 and BL60, there was a spatial distinction between their connectivity patterns (Figure 4 and Figure 5ab), implicating functional specificities of these two visual acupoints during the late phase of acupuncture.

### ICA results

ICA analysis showed that nine regions were included in the visual network of the RUN 31 period, i.e. the right lingual gyrus and right cuneus, thalamus, left inferior frontal gyrus, bilateral temporal-occipital gyrus, bilateral fusiform and PCC/pC.

### CNA results

**Figure 6:**
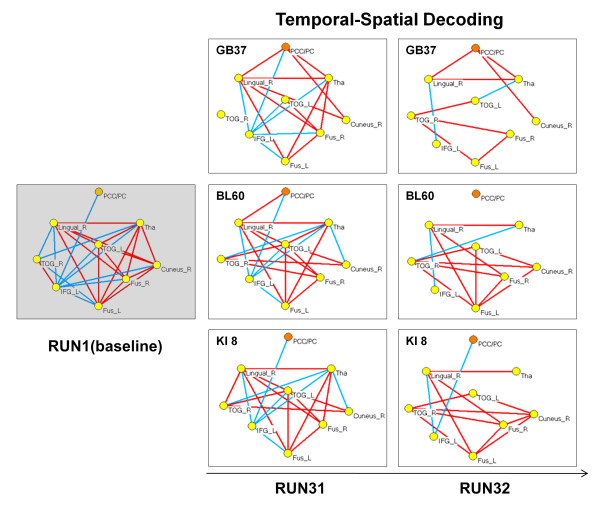
**The results from graphic theory-based CNA (see **Methods**), showing the topological and functional maps of the visual RSN (consisting of the yellow nodes) and its dynamic interactions with the PCC/pC (the red node) as a system encoding/decoding hub with respect to three different acupoints (two visual acupoints: GB37 and BL60, and a non-visual acupoint KI 8)**. The red lines referred to the significant (*p *< 0.05, see the **Methods **for details) positive functional connections among the visual function-related network, and the blue lines the significant negative connections.

To further detect how the sustained effects of acupuncture modulate the CNS response, the ROI-based effective CNA was employed to show that peripheral stimulation at an acupoint would induce corresponding activity changes in a specific brain network (Figure 6). In the last step, ICA derived nine nodes. As our hypothesis indicated, PCC/pC was regarded as the deciphering center. In this way, the analyses were focused on the connections between ICA-derived nine nodes as well as the connectivity changes within the network. Note that almost no changes in the modulation of the visual RSN were found during RUN 31 for KI8, although slight changes were shown during RUN 32 (Figure 6). Based on this intrinsic visual RSN (i.e. represented by the KI8 network since no change was observed during Run 31), our analyses revealed differential dynamic modulations (regarding the number of connections at each of the network nodes and temporal changes in these connections across the two runs) of the visual function-related network after stimulation at these three acupoints respectively (Figure 6).

The interactions between the PCC/pC and the visual RSN were through their connections with the occipital nodes (the lingual gyrus and cuneus), thalamus (THA), and inferior frontal gyrus (IFG). A higher level of salient positive correlations (red lines) between the PCC/pC and the visual RSN was found after acupuncture stimulation at GB37 compared to BL60. Furthermore, the PCC/pC was positively correlated with the occipital cortices through the lingual gyrus only during RUN 31 after acupuncture at BL60. No such positive correlations between the PCC/pC and occipital cortices were detected after stimulation at KI8 [14].

## Discussion

The present study used fMRI to reveal the CNS signatures of acupoint specificities. Without scanning brain activities, the verification of acupuncture effects in patients could only rely on their *DeqiDeqi *sensations and clinical outcome; such results might not always be stable and consistent [9,10,24,43]. Our behavioral measurements have shown no difference in the subjective ratings of various *DeqiDeqi *sensations with respect to stimulations at the three acupoints, which indicate that no acupoint specificity can be detected explicitly. Although *Deqi *is an essential step during acupuncture, the brain representation of explicit sensations during needling manipulation may not explain specific healing effects of acupuncture or its clinical relevance. Nevertheless, most previous fMRI studies focusing on the acute stage of acupuncture might merely reveal its subjective or placebo-like effects [9,44], rather than being pertinent to functional specificities of acupuncture. In the present study using a new experimental paradigm to focus on the post-effects of acupuncture (Figure 1), we have demonstrated that the functional specificities of acupuncture may be mediated through brain pathways independent of the concurrent subjective sensations (Figure 6; see below).

An interesting finding here is the converging brain response at PCC/pC when different acupoints were stimulated (Figure 3), which is consistent with the common effects found during the acute phase of acupuncture [8-10,44]. Besides, the overlapped PCC/pC response provided a shared background for comparing functional connectivity patterns so as to assess acupoint-brain specificities. Our studies showed distinct brain modulations during and after acupuncture stimulation, in which the PCC/pC was differentially interacting with the occipital lobe (Figure 4 and Figure 5). Such direct comparisons showed the effects at functionally distinct regions, which were highly specific to the visual acupoints. While an apparent opposite pattern was found in the time courses from these brain regions, which might implicate diverse neural dynamics, the mechanism behind the temporal responses has yet to be elucidated.

Results from CNA further demonstrated how the PCC/pC interacted with a specific functional network (i.e., the visual RSN), suggesting that the PCC/pC might serve as an information hub [45] to drive the intrinsic visual network and implement specific brain function after acupoint stimulation (Figure 6). The PCC/pC is a core region involved in the so-called default-mode network (DMN) [33,46], which can be modulated by external tasks or stimuli [47]. Previous studies have shown that peripheral stimulation at an acupoint may mobilize the DMN to mediate its action [16,48]. Therefore, the resting state fMRI analysis of the intrinsic functional networks (including the DMN) [49,50] is critical to form our hypothesis that peripheral acupoint stimulation is encoded intrinsically in the brain via interaction between the PCC/pC and specific RSNs. Note that the graphic theory-based CNA (see Methods) was used here as a hypothesis-driven approach, and our hypothesis was formulated by pre-defining the intrinsic visual RSN as a basic module [51] in the CNA for testing the functional specificities of acupuncture.

The investigation into the question how acupuncture over visual and non-visual acupoints may modulate the intrinsic visual network is essential to the study of acupuncture specificities. The results from CNA revealed that the PCC/pC interacted differentially with the functional network across these different acupoints, indicating that the PCC/pC might convey distinct modulations after needling. Positive correlations between the PCC/pC and other nodes in the visual RSN were found for both the GB37 and BL60 groups, indicating enhanced functional connectivity between the PCC/pC and visual RSN. However, the non-visual acupoint group showed an opposite pattern via negative correlations. Moreover, the disparate patterns of modulations endured, indicating a stronger sustained effect of acupuncture in the GB37 and BL60 groups relative to the KI8 group. These results demonstrated that acupuncture stimulation at various acupoints induced corresponding activity changes in the functionally specific network, i.e. the visual RSN.

Findings of delayed correspondence between the visual acupoints (but not the non-visual acupoint) and the intrinsic visual RSN (Figure 6) purport a temporal-spatial encoding mechanism underlying the functional specificity of acupuncture. Similar to the functional mapping of sensory and motor homunculi, the neural encoding of peripheral acupoints can be revealed by the analysis of corresponding brain networks. However, it would be naïve and arbitrary to consider the acupoint-brain specificity equivalent to the well-established sensory homunculus, i.e. stimulating the skin activates the brain selectively, since acupuncture effects could last much more extensively and profoundly [8,28]. It was also different from the previous fMRI study of somatosensory encoding during the acute phase of acupuncture [31], where our complex network analysis took into account the non-acupoint manipulation as the control as well the multi-level encoding processes, including both the intrinsic encoding by the RSNs and the functional encoding by modulation of the RSNs even after the external stimuli were terminated. Furthermore, the sustained phase of acupuncture may construct a deciphering process, in which the delayed effects are dispensed into distinct brain networks [11,12,15,17]. In other words, the CNS may encode differentially after being triggered by acupuncture needling at different acupoints, and then the corresponding functional networks may mediate specific treatment effects of acupuncture. Although the visual acupoints were traditionally defined according to their clinical relevance, to validate their therapeutic effects would be beyond the scope of the present study as it was conducted only on healthy subjects.

In sum, we have provided an integrated view to explain functional specificities of acupuncture and how stimulation of peripheral acupoints and their corresponding neural substrates interact with each other. Our fMRI results demonstrated that the PCC/pC might serve as an encoding/decoding hub interacting with distinct brain networks when different visual or non-visual acupoints were stimulated, and that both the needling sensation and the following neural cascades contributed to the overall effect of acupuncture. Moreover, these findings implicate that acupuncture stimulation at different acupoints may achieve differential brain modulations and bear the corresponding healing effects through dynamic reconfiguration of the neural networks.

## Conclusion

The present study used fMRI to reveal CNS signatures of acupoint specificities. We employed a CNA approach to test the hypothesis that acupuncture stimulation at an acupoint would induce corresponding activity changes in one or more intrinsic or resting state brain networks. Built upon the sustained effect of acupuncture and its time-varying characteristics, we constructed a dynamic encoding system with a hub at the posterior cingulate cortex and precuneus (PCC/pC). This study provided an integrated view to explore the functional specificity of acupuncture, in which both the needling sensation and the following neural cascades may contribute to the overall effects of acupuncture through dynamic reconfiguration of complex neural networks.

## Methods

### Subjects

Forty five healthy, acupuncture naïve, right-handed Chinese subjects [24 males; ages of 24.2 ± 2.9 years (mean ± SD)] participated in the fMRI experiment. Subjects were all acupuncture naïve, and right-handed according to the Edinburgh Handedness Inventory. They had no history of neurological or psychiatric disorders and all had been refrained from alcohol consumption or any medication in the previous 48 hours. After a complete description of the study was given to all subjects, written informed consent was obtained; the research protocol was approved by the West China Hospital Subcommittee on Human Studies. The experiment was also conducted in accordance with the Declaration of Helsinki.

### Experimental paradigm

Three acupoints on the left leg were selected for stimulation in the experiment according to their potential effects on human brain functions [10,13]: two *visual *acupoints, Gall Bladder 37 (GB37 or *Guang Ming*) and Bladder 60 (BL60 or *Kun Lun*), and one *non-visual *acupoint, Kidney 8 (KI8 or *Jiao Xin*). GB37 is located 5 cm superior to the prominence of the lateral malleolus, BL60 is located in the indent midway between the external malleolus and the calcaneus tendon of the lateral ankle and KI8 is posterior to the medial border of the tibia (Figure 1). 45 subjects were equally divided into three groups, with each subject pseudo-randomly assigned to one of the three acupoints for stimulation. The effect of gender, ages and order effect of conditions as counterbalanced. All participants were blinded to in the group selection process.

A non-repeated event-related (NRER) paradigm [11] was employed. The overall fMRI paradigm consisted of three functional scans or runs lasting about 30 minutes: a pre-stimulation resting scan (treated as the baseline), a functional scan while acupuncture (needling) manipulation was applied, and a post-stimulation resting scan (Figure 1). More specifically, before acupuncture stimulation, each subject underwent a baseline fMRI scan lasting 6 minutes. Then, the needle was inserted into one of the three acupoints (i.e. GB37, BL60, or KI8) according to group assignment. After 1 minute of subject adaptation, the subject underwent a second functional scan lasting 6 minutes while electroacupuncture (EA) stimulation, 2 Hz and 2-3 mA, was applied. The needle was then withdrawn. To monitor the post-stimulation effect, the subject underwent a third fMRI scan lasting 12 minutes. During the entire experiment, subjects wore a blindfold and were instructed to remain calm with their eyes closed. The needles were 0.18 mm in diameter and 40 mm in length. The needles were inserted into the skin at a depth of 1.0 to 1.5 cm. The acupuncture manipulation was conducted by a professional acupuncturist.

### Psychophysical measurement

Immediately after EA stimulation ceased, participants were asked to quantify their subjective sensations of *Deqi *based on a verbal analog scale [9,40]. The sensations were described on the MGH Acupuncture Sensation Scale, including aching, soreness, pressure, heaviness, fullness, warmth, coolness, numbness, tingling, throbbing, dull or sharp pain and one blank row for the subjects to add additional descriptions if the above descriptors failed to embody the sensations they experienced during stimulation [40]. The intensity of each sensation was measured on a scale from 0 to 10 (0 = no sensation, 1-3 = mild, 4-6 = moderate, 7-8 = strong, 9 = severe and 10 = unbearable sensation). ANOVA was employed to assess whether or not there were any differences in *Deqi *sensation intensities among groups. Because sharp pain was considered an inadvertent noxious stimulation, the participants who experienced sharp pain (greater than the mean by more than two standard deviations) were excluded from further analysis.

### Data acquisition and analysis

A foam pillow was used to facilitate in keeping participants' heads motionless during scanning. The fMRI experiment was performed on a 3.0 Tesla GE Signa MR whole body system. The blood oxygenation-level dependent (BOLD) contrast-based images (32 sagittal slices, 5 mm thick with no gaps) were collected using a single-shot gradient recalled echo planar imaging (EPI) sequence (TR/TE = 2000 ms/30 ms, flip angle = 80°, field of view = 240 × 240 mm^2^, matrix size = 64 × 64, in-plane resolution = 3.75 × 3.75 mm^2^_. A set of T_1_-weighted high-resolution structural images (TR/TE = 5.7 ms/2.2 ms, flip angle = 7°, FOV = 256 × 256 mm^2^, matrix size = 256 × 256 mm^2^, in-plane resolution 1 × 1 mm^2^, slice thickness = 1 mm) were collected for anatomical localization.

Three datasets were created, i.e. RUN 1, RUN 31 and RUN 32. Images in RUN 1 were truncated from a pre-stimulation scan session, consisting of volumes from 0.5 to 5.5 min. To investigate the dynamic characteristics of the sustained effects of acupuncture [8], the images of RUN 3 were divided into two datasets: images in RUN 31 were acquired from a post-acupuncture session, consisting of volumes from 0.5 to 5.5 min and images in RUN 32 were acquired from the same session, consisting of volumes from 6 to 11 min. All datasets were intensity scaled to yield a whole brain mode value of 1,000. The preprocessing steps were carried out using statistical parametric mapping (http://www.fil.ion.ucl.ac.uk/spm/). The images were realigned to the first image. The subject was excluded for further analysis if the head motion was more than 1 mm or more than 1 degree in any direction. The T1-weighted high resolution image volume was co-registered to the mean EPI image and spatially normalized to the Montreal Neurological Institute T1-weighted template, which was chosen from an SPM5 software package, and re-sampled at 3 mm × 3 mm × 3 mm.

#### ANOVA

First, we had to examine whether or not all three groups had an equivalent baseline. Three datasets were analyzed with ANOVA and the results showed that there was no statistical difference between groups (*p *< 0.001, uncorrected), i.e. pre-stimulation resting state.

#### Discrete cosine transform (DCT)

DCT, as a pattern recognition method, was applied to detect spatial distributions of any signal changes in specific band frequencies [15,34]. The DCT analysis was performed using SPM5 (http://www.fil.ion.ucl.ac.uk/spm/). To test for temporal effects, the RUN 31 dataset was spatially smoothed with a 6 mm full-width-at-half maximum (FWHM) Gaussian kernel for the discrete cosine transform (DCT) analysis. Here, the discrete cosine basis set contained 60 regressors spanning the frequencies range from 0 to 0.1Hz, and the group results of each condition were obtained by computing the F-contrasts for the effect of signal fluctuation in the range of 0.01 - 0.1Hz (*p *< 0.001, corrected for multiple comparisons) [34].

#### Conjunction analysis

The group-level results of each group were converted as a binary map. Then, the conjunction analysis was employed to detect inter-group similarities of the spatial distribution patterns using the masks from the last step [33]. As a result, PCC/PC was selected as the ROI for the functional connectivity analysis.

#### Functional connectivity analysis

This procedure was performed on RUN 1, RUN 31 and RUN 32 of each group. The functional connectivity maps were created by computing the correlation coefficients between the BOLD time course of the ROI and that of all of the other brain voxels. The correlation coefficients were converted to a normal distribution by Fisher's Z transform. The intra-group analysis and inter-group analysis were conducted using group *t*-tests. Then, the second level analysis was preformed to detect the variations between the post-stimulation state and the resting state using a paired *t*-test. The specific processing steps were: (I) reducing the global signal correlation, where all data sets were first processed with a bandpass filter (0.01~0.1Hz) and then spatially smoothed with a 6 mm FWHM Gaussian kernel; (II) removing several spurious variances along with their temporal derivatives with linear regression, the six head motion parameters and the signals from a region centered in the white matter and a region centered in the cerebrospinal fluid; (III) correlation maps were created by computing the correlation coefficients between the BOLD time course from the seed region and the BOLD time course from all of the other brain voxels; and (IV) correlation coefficients were converted to a normal distribution by Fisher's Z transform. Then, the intra-group analysis and the inter-group analysis were conducted.

The 2^nd ^level analysis was performed using the random effects analysis based on the one sample *t*-test for the two periods after EA stimulation, based on the paired *t*-test used to detect the variations between the post-stimulation state and the resting state. Finally, we investigated the acupuncture specificity among the three acupoints based on the two sample *t*-test.

#### Independent component analysis (ICA)

ICA was used to determine an intrinsic RSN related to the visual function of the RUN 1 dataset [51,52]. This paper focused on the modulation of vision-related acupoints over the visual cortex, so that the mask of the occipital lobe was used to define the functional map. An anatomical template of the occipital lobe was defined using the AAL ROI template (anatomically defined by hand on a single brain region matched to the MNI/ICBM templates) [53]. Then, the smoothed images from the RUN 1 datasets from all three conditions were arranged into Group ICA of the fMRI Toolbox (GIFT, http: http://icatb.sourceforge.net) [54] for ICA analysis. The processing in GIFT includes initialization, PCA reduction and ICA extraction. ICA was performed using a neural network algorithm (Infomax) that attempted to minimize mutual information of the network outputs [55,56]. The mean ICs of all subjects and the ICs for each subject were obtained from Group ICA separation and back reconstruction [54]. Then, a frequency filter was applied to remove those components in which a high-frequency signal (>0.1 Hz) constituted 50% or more of the total power in the Fourier spectrum [53]. Next, the template defined above was used to select the 'best-fit' of the remaining low-frequency components in each subject[53]. A one sample *t*-test was performed to determine the intrinsic RSN related to visual function.

#### Functional connectivity by CNA

To investigate the sustained effects of acupuncture and its modulation on visual-related functional networks, CNA was adopted [35,36]. The method has the capability of evaluating the strength as well as the temporal and spatial patterns of interactions in human brains. It defines a graph as the combination of a set of nodes (brain regions) and edges (functional connections). In this way, the graphic visualization provides strong implications of functional connectivity among brain regions [38,39].

Independent component analysis resulted in nine regions in the occipital lobe for further graphic theory-based CNA. The nine visual-related nodes were taken into complex network mode analysis during RUN 31 and RUN 32. The regional mean time series were extracted by averaging the fMRI time series of all of the voxels in each of these ROIs over the entire brain. Partial correlation was used to construct undirected graphs. We first had to relate the threshold () with the partial correlation coefficient (*r_ij_*), so that each statistically significant connection could be represented as an undirected edge if *r_ij _*exceeds . Thus, edges between regional nodes presented the whole brain functional network. Finally, a graph of 9 nodes and edges (functional connections) were defined.

## List of abbreviations

PCC/pC: the posterior cingulate cortex and precuneus; CNS: central nervous system; NRER: non-repeated event-related; fMRI: functional magnetic resonance imaging; RSN: resting state network; DCT: discrete cosine transform; CNA: complex network analysis; ROI: region of interest; THA: thalamus; IFG: inferior frontal gyrus; MTG: middle temporal gyrus; FUS: fusiform; _R: right; _L: left; DMN: default-mode network; SD: standard deviation; EA: electroacupuncture; EPI: echo planar imaging; BOLD: blood oxygenation-level dependent; FWHM: full-width-at-half maximum; GIFT: Group ICA of the fMRI Toolbox.

## Competing interests

The authors declare that they have no competing interests.

## Authors' contributions

WQ carried out the experiment and wrote the manuscript. LJB participated in the data processing and provided assistance in writing the manuscript. JPD participated in the design and coordination of this study. MHD participated in the design of this study. PC performed the entire acupuncture procedure. JXL participated in the design and coordination of this study. PL participated in the design of this study. KY participated in the design of this study. BXZ performed the entire acupuncture procedure. JBS participated in the data processing and provided assistance in writing the manuscript. QYG participated in the design and coordination of this study. JT participated in the design and coordination of this study. YJL provided fMRI methodology in the study and assisted in writing the manuscript. All authors read and approved the final manuscript.
